# Dietary calcium intake does not meet the nutritional requirements of children with chronic kidney disease and on dialysis

**DOI:** 10.1007/s00467-020-04571-x

**Published:** 2020-05-08

**Authors:** Louise McAlister, Selmy Silva, Vanessa Shaw, Rukshana Shroff

**Affiliations:** 1 Great Ormond Street Hospital for Children NHS Foundation Trust, London, WC1N 3JH UK; 2 University College London Institute of Child Health, London, UK; 3 University of Plymouth, Plymouth, UK

**Keywords:** Calcium, Diet, Bones, Chronic kidney disease, Dialysis

## Abstract

**Background:**

Adequate calcium (Ca) intake is required for bone mineralization in children. We assessed Ca intake from diet and medications in children with CKD stages 4–5 and on dialysis (CKD4–5D) and age-matched controls, comparing with the UK Reference Nutrient Intake (RNI) and international recommendations.

**Methods:**

Three-day prospective diet diaries were recorded in 23 children with CKD4–5, 23 with CKD5D, and 27 controls. Doses of phosphate (P) binders and Ca supplements were recorded.

**Results:**

Median dietary Ca intake in CKD4–5D was 480 (interquartile range (IQR) 300–621) vs 724 (IQR 575–852) mg/day in controls (*p* = 0.00002), providing 81% vs 108% RNI (*p* = 0.002). Seventy-six percent of patients received < 100% RNI. In CKD4–5D, 40% dietary Ca was provided from dairy foods vs 56% in controls. Eighty percent of CKD4–5D children were prescribed Ca-based P-binders, 15% Ca supplements, and 9% both medications, increasing median daily Ca intake to 1145 (IQR 665–1649) mg/day; 177% RNI. Considering the total daily Ca intake from diet and medications, 15% received < 100% RNI, 44% 100–200% RNI, and 41% > 200% RNI. Three children (6%) exceeded the National Kidney Foundation Kidney Disease Outcomes Quality Initiative (KDOQI) upper limit of 2500 mg/day. None with a total Ca intake < RNI was hypocalcemic, and only one having > 2 × RNI was hypercalcemic.

**Conclusions:**

Seventy-six percent of children with CKD4–5D had a dietary Ca intake < 100% RNI. Restriction of dairy foods as part of a P-controlled diet limits Ca intake. Additional Ca from medications is required to meet the KDOQI guideline of 100–200% normal recommended Ca intake.

**Figure:**
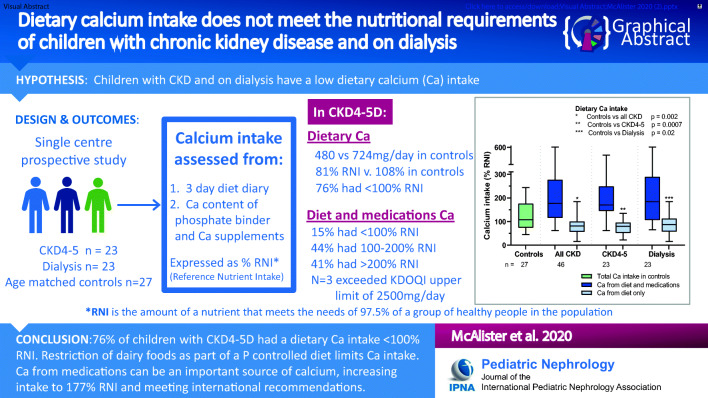
Graphical abstract

**Electronic supplementary material:**

The online version of this article (10.1007/s00467-020-04571-x) contains supplementary material, which is available to authorized users.

## Introduction

Ensuring adequate calcium (Ca) intake, along with maintaining vitamin D status and limiting dietary phosphate (P) exposure, is central to the dietary management of chronic kidney disease mineral and bone disorder (CKD-MBD) in children [1]. A low Ca intake is especially of concern in children with chronic kidney disease (CKD) who require Ca for bone accrual and growth and to prevent fractures [2], 90% of peak bone mass being accrued by 18 years of age [3]. Conversely, excess Ca may lead to coronary artery calcification which increases cardiovascular risk, the leading cause of death in this population [4, 5].

A large prospective cohort reported that children with even early CKD have a 2–3-fold higher fracture rate compared with that of their healthy peers [2]. The use of Ca-based P-binders was protective against fractures. Vascular calcification has also been identified early in CKD [6], and clinicians are increasingly favoring non-Ca-based P-binders to reduce Ca load, particularly for the adult dialysis population [7]. A study in young adults on dialysis [8] reported that the Ca intake from P-binders was nearly twice as high in those with coronary artery calcification compared with those without; no estimate of dietary Ca intake was made. However, extrapolating adult studies to pediatrics are not appropriate as children need a positive Ca balance [9], particularly at periods of rapid growth. Kidney Diseases Improving Global Outcomes (KDIGO) and the National Institute for Health and Care Excellence (NICE) hyperphosphatemia guidelines [10, 11] recommend that a Ca-based binder be used as first-line treatment in controlling hyperphosphatemia in children in order to meet the Ca requirements of the growing skeleton. International recommendations for Ca intake in children with CKD [1, 12] suggest that the total Ca intake from diet and medications should be 100–200% of normal requirements, apart from exceptional circumstances.

The aim of this study was to assess the Ca intake from food, formula feeds (FF; including infant milks, oral nutritional supplements and enteral tube formulas) and medications in children with CKD4–5D and controls. We quantified the Ca intake from diet, P-binders, and Ca supplements, expressed as a percentage of the UK Reference Nutrient Intake (RNI) [13], and compared this with the recommended Ca intake suggested by KDOQI [1]. The foods primarily contributing to Ca intake within this CKD population were identified and contextualized by comparison with controls and data from the UK national food consumption surveys [14, 15].

## Methods

This is a single-center, prospective cross-sectional study that is part of a larger project assessing Ca balance in children and young adults with CKD [16]. Children from birth to 18 years of age with CKD4–5D attending renal out-patient clinics at Great Ormond Street Hospital were invited to participate. Age-matched controls were selected from siblings and friends of patients as well as children attending dermatology, ear, nose, and throat or plastic surgery minor operating lists at our hospital. All participants and/or their caregivers provided informed written consent and assent appropriate for their age. The study was approved by the research ethics committee and the NHS Health Research Authority.

Routine serum biomarkers were measured in CKD subjects at their clinic visit or prior to a mid-week hemodialysis session, and at the planned study visit or routine pre-operation assessment clinic for controls. These included serum total Ca, ionized Ca, P, magnesium, bicarbonate, intact parathyroid hormone (PTH), 25-hydroxyvitamin D [25(OH)D], and alkaline phosphatase. PTH concentrations were measured by the Immulite 2000 Intact PTH immunoassay (Siemens Healthcare Diagnostics, Frimley, Surrey, UK). 25(OH)D concentrations were analyzed by isotope-dilution liquid chromatography-tandem mass spectrometry (Waters Xevo TQ-S, Waters UK, Elstree, Herts, UK). Ca-based P-binders and sevelamer were prescribed as per NICE hyperphosphatemia guidelines [11].

Diet diaries for 3 consecutive days (including one weekend day) were completed by each child or their parents/caregivers, following instruction by the research nurse (SS). Participants were encouraged to describe the type, amount, and cooking method of all foods and drinks consumed, including packages and labels from manufactured food items where possible. The volume and composition of FF (oral and enteral) were recorded. To reduce participant burden and simplify day to day documentation, the full description of items that were repeatedly consumed was recorded once at the start of each diary (e.g., cow’s milk, semi-skimmed; bread, wholemeal thick-sliced). To ensure consistency in interpretation, one dietitian (LM) analyzed the diaries and (where necessary) converted food portions into weights using the UK Food Standard Agency Food Portion Sizes handbook [17]. Ca content was assessed with a software program, CompEat Pro (Nutrition Systems; www.compeat.co.uk), which uses the reference UK food nutrient analysis database [18]. Manufacturers’ nutritional compositional data was used for FF. Calcium in drinking water (tap or mineral) was not included in the analysis, and the volume of water consumed was not recorded.

The mean daily dietary Ca intake from each completed 3-day diary was assessed and compared with the relevant age and sex UK RNI (defined as the amount of a nutrient that is enough to meet the needs of 97.5% of a group of healthy people in the population) [13].

The median daily dietary Ca intake for 4 groups of children were compared: controls, those with CKD4–5, CKD5D (on dialysis), and all children with CKD4–5D. This was expressed as mg Ca/day, mg Ca/kg body weight/day and as a percentage of RNI. The relative contribution of different food groups to dietary Ca intake was calculated for controls and all groups of children with CKD. The distribution was then compared with data for children from a UK cross-sectional survey of a representative sample of the general population, the National Diet and Nutrition Survey (NDNS) [14, 15].

The Ca from Ca-based P-binders and Ca supplements was quantified based on prescribed amounts, and the total combined daily Ca intake from diet, FF, and medications was calculated for all children as described above. Dietary P consumption was also assessed from the diet diaries, but it is acknowledged that this does not reliably reflect total P intake, as the amount from P-containing food additives cannot be quantified [19, 20].

### Statistical analysis

Distributions of all variables were assessed for normality. Descriptive statistics are presented for demographic and clinical characteristics of the study cohort. Continuous variables are presented as medians and interquartile ranges (IQR), whereas frequencies and percentages are used for categorical variables. Anthropometric indices are expressed as standard deviation score (SDS) for age and sex. Comparisons of continuous variables between groups were performed using Mann-Whitney or Kruskal-Wallis tests as appropriate. Independent *t* testing was used for between group analysis, and for group comparison of categorical variables, the Pearson chi square test or Fishers exact *t* test was used. The relationships between continuous variables were assessed with Spearman’s correlation coefficient. Statistics were calculated using SPSS Statistics 24.0 (IBM Corporation) and graphs constructed using Graph Pad Prism (version 8.3). A *p* value of < 0.05 was considered statistically significant, and two-sided testing of the hypothesis was used for all tests where appropriate.

## Results

Baseline characteristics of the study population are described in Table 1. The groups were matched for age, sex, and ethnicity, but, as expected, the median height and weight SDS of CKD4–5D children was significantly lower than that of the controls. The median BMI SDS for CKD4–5D and controls were not significantly different. Ninety-two percent of participants completed a 3-day diet diary and 100% completed 2 days.

**Table 1:** Demographics, anthropometry and routine clinical measures for the study population and controls

	CKD4–5 (*n* = 23)	CKD5D (dialysis) (*n* = 23)	All CKD (CKD4–5D) (*n* = 46)	Controls (*n* = 27)
Age (years)				
Median (IQR)	10.2 (6.5–12.5)	6.9 (2.5–14.1)	9.1 (4.8–14.1)	10.1 (4.8–14.7)
Sex (F, %)	7 (30%)	13 (57%)	20 (43%)	15 (56%)
Underlying kidney disease				
CAKUT/cystic/glomerulonephritis/others	19/3/1/0	13/1/3/6	32/4/4/6	-
Ethnicity				
Caucasian/Asian/African/Arabic/mixed	21/2/0/0/0	9/9/4/1/0	30/11/4/1/0	18/5/1/0/3
eGFR* (mL/min/1.73m^2^) median	16 (11–23)	-	-	120 (110–128)
Dialysis				
Mode (PD/HD)	-	8/15	-	-
Time on dialysis (years)	-	1.21 (0.4–3.3)	-	-
Anthropometry				
Weight SDS	− 0.2	− 1.8	− 1.0	0.4
Height SDS	− 0.8	− 2.0	− 1.6	0.4
BMI SDS	0.6	− 0.1	0.4	0.3
Vitamin D analogues (*n*, %)				
Colecalciferol	7 (30%)	12 (52%)	19 (41%)	-
Alfacalcidol	22 (96%)	20 (87%)	42 (91%)	-
Both	6 (26%)	11 (48%)	17 (37%)	-
Vitamin D status**				
Median 25(OH)D (nmol/L)	85 (72–146)	148 (70–115)	111 (72–172)	62 (41–76)
< 50 (%)	19	17	18	33
50–75 (%)	24	17	21	33
> 75 (%)	57	66	61	33
Serum calcium (mmol/L)	2.48 (2.43–2.54)	2.49 (2.39–2.55)	2.49 (2.41–2.56)	2.33 (2.29–2.41)
Serum ionized calcium (mmol/L)	1.21 (1.17–1.26)	1.22 (1.18–1.29)	1.20 (1.17–1.24)	1.19 (1.13–1.22)
Serum phosphate (mmol/L)	1.46 (1.32–1.46)	1.7 (1.25–1.90)	1.59 (1.29–1.80)	1.46 (1.37–1.64)
PTH (pmol/L)	6.9 (3.2–16.5)	21.9 (8.3–44.3)	12.2 (4.2–32.1)	4 (2.4–7.18)
Alkaline phosphatase (U/L)	179 (133–242)	247 (174–350)	201 (154–282)	181 (120–292)***

*Schwartz formula [39]

**Vitamin D status, < 50 nmol/L, deficient; 50–75 nmol/L, insufficient; > 75 nmol/L, sufficient [40]

***n = 18

*IQR* interquartile range, *CAKUT* congenital anomalies of kidney and urinary tract, *eGFR* estimated glomerular filtration rate, *PD* peritoneal dialysis, *HD* hemodialysis, *SDS* standard deviation score, *BMI* body mass index

### Dietary Ca intake

The median dietary Ca intake in CKD4–5D was 480 (IQR 300–621) vs 724 (IQR 575–852) mg/day in controls (*p* = 0.00002), providing 81% vs 108% RNI for age (*p* = 0.002). This equates to 19 (IQR 14–40) vs 18 (IQR 13–45) mg Ca/kg/day in CKD4–5D vs controls. Accounting for the growth deficit in CKD4–5D (median weight and height SDS − 1.0 and − 1.6 respectively), the percentage intake was 80% (IQR 58–118) vs 108% (IQR 75–176) of RNI for height age (*p* = 0.008).

**Fig. 1:**
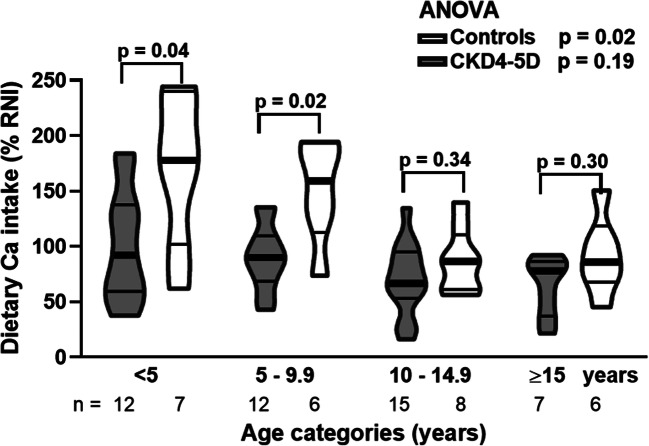
Dietary Ca intake in children with CKD4–5D and controls for different age groups. Violin plots with the thick bar representing the median and thinner lines representing inter quartile range. Children are categorized into four age groups. The ANOVA compares Ca (% RNI) in children of all age groups (for controls *p* = 0.02; for CKD4–5D *p* = 0.19)

Seventy-six percent of children with CKD4–5D received < 100% RNI for Ca. Two of the controls (but none of those with CKD4–5D) had a dietary Ca intake exceeding 2 × RNI for age. Comparing dietary Ca intake in CKD4–5D with controls by age, children under 10 years with CKD4–5D had significantly lower dietary Ca as % RNI, but this difference was not significant in those over 10 years (Fig. 1). Dietary Ca intake in CKD4–5D and controls expressed as mg/kg/day, showed a negative correlation with age (R^2^ = − 0. 45 vs *R*^2^ = 0.66. respectively; Supplementary Fig. 1). There was no difference in the Ca intake (% RNI) in those with CKD4 vs CKD5 (*p* = 0.06) or between CKD4–5 and CKD5D (*p* = 0.42).

Twelve out of 23 (52%) children on dialysis were exclusively FF, achieving 89% RNI Ca, which is comparable with that of controls (*p* = 0.08). Twelve (26%) of children with CKD4–5D received partial FF, that provided 31 (23–40)% of dietary Ca intake or 28 (13–51)% RNI. Those children with CKD4–5D not exclusively FF had a lower median Ca intake (79% RNI), and this was significantly lower than controls (*p* = 0.0009).

### Main dietary sources of Ca

**Fig. 2:**
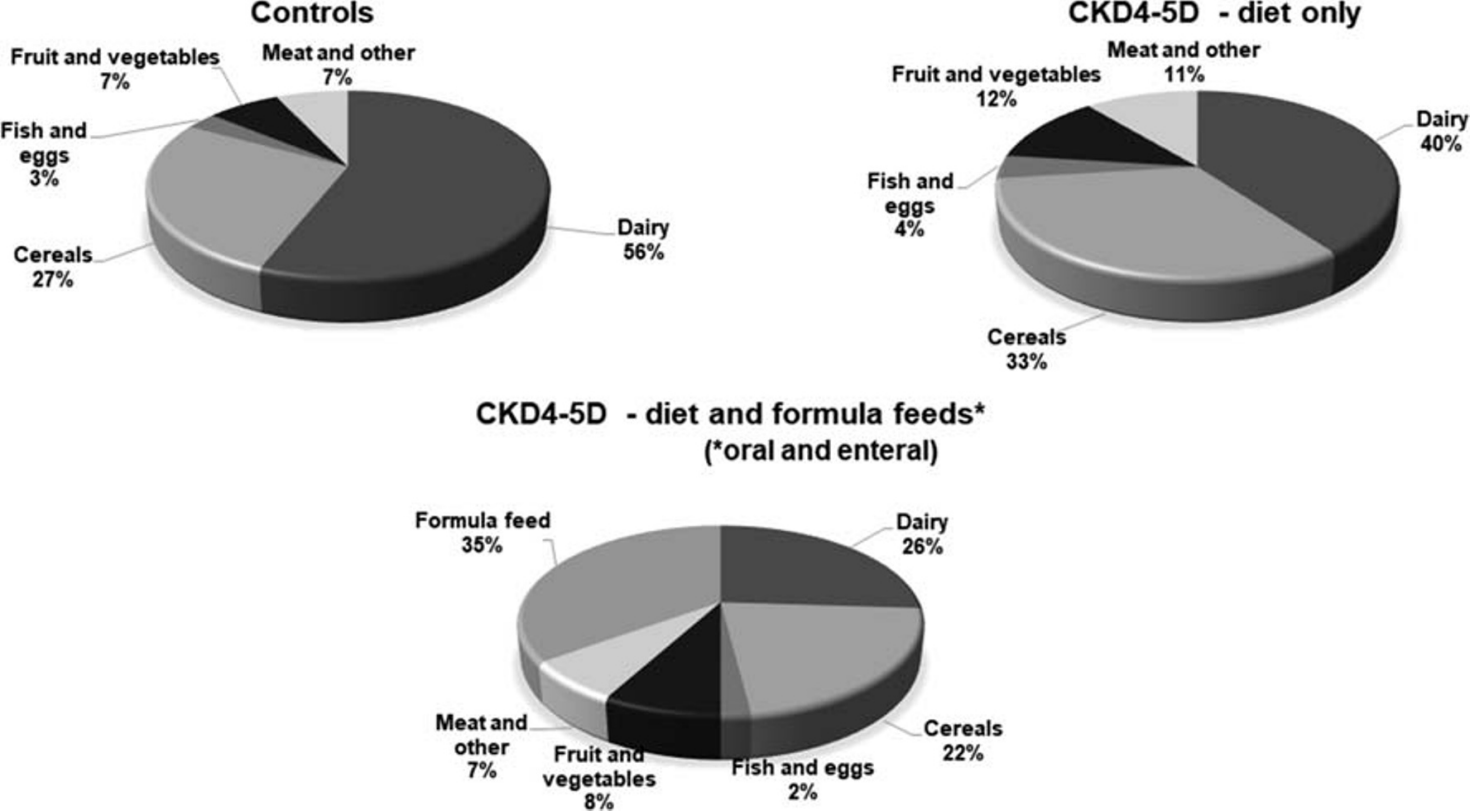
Main food groups contributing to Ca intake (% total intake per day), in controls and CKD4–5D. Ca contributed from diet alone and diet plus formula feeds are shown separately

In controls, dairy foods and cereals (including grains made into pasta, rice, breads, breakfast cereals and biscuits) were the main sources of dietary Ca, contributing 56% and 27% respectively of intake, similar to that reported for the UK population from the NDNS [14, 15] (Fig. 2). Excluding those who were exclusively FF, children with CKD4–5D had a significantly lower proportion from dairy foods (40%) and an increased contribution from cereals (33%). When the Ca provided by FF was included, the contribution from dairy foods further reduced to 26% of total dietary intake.

**Fig. 3:**
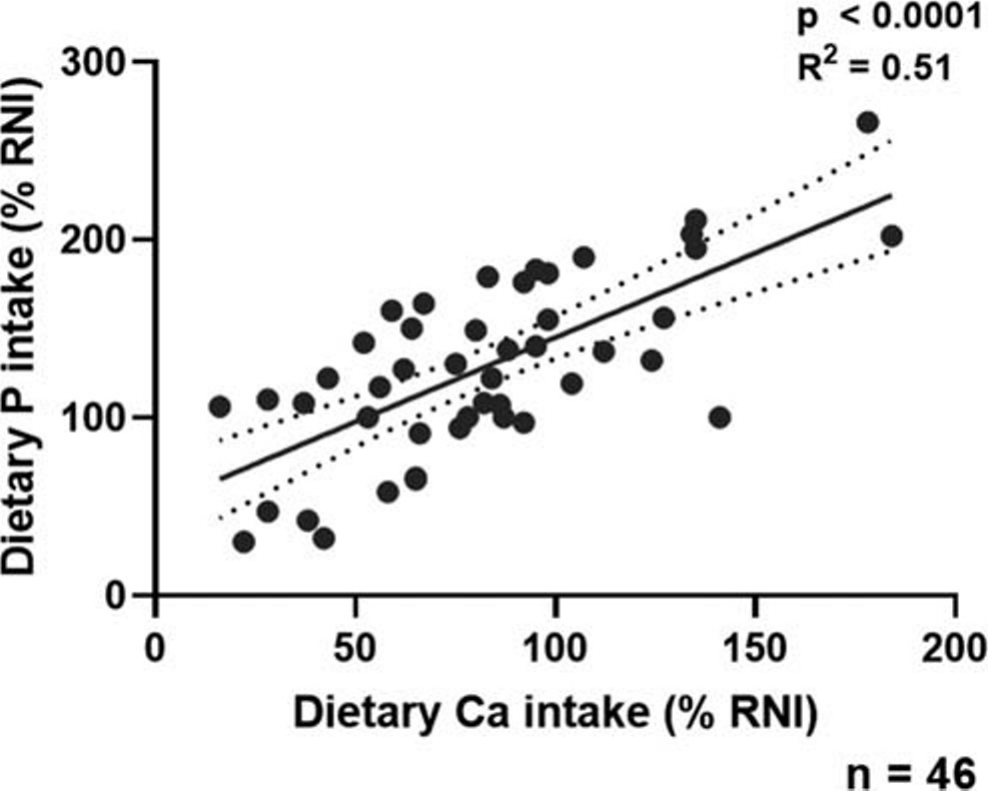
Relationship between dietary Ca and P intake in children with CKD4–5D. The linear regression with 95% confidence intervals is shown

The daily P intake and contribution of different foods to P intake are shown in Supplementary Tables 1 and 2. The significant contribution of dairy foods and cereals to both Ca and P intakes is reflected by the close correlation between Ca and P intake (as a % RNI) in the children with CKD4–5D (*p* < 0.0001, *R*^2^ = 0.51; Fig. 3).

### All CKD

#### Total Ca intake from diet and medications

80% (37/46) of children with CKD4–5D were prescribed Ca-based P-binders, including 2 children who received Ca-based P-binders and sevelamer (Table 2). No child was on a non-Ca-based P-binder alone. Seven children (15%) needed Ca supplements, and 4 (9%) were on both Ca supplements and Ca-based P-binders. Three children requiring Ca supplements also received a high dialysate Ca (1.75 mmol/L compared with the standard 1.25 mmol/L; 2 on HD and 1 on PD). There was no correlation between age and the type of Ca-based P-binder used.

**Table 2:** Use of phosphate binders, calcium supplements, and phosphate supplements

Medications	All CKD (*n* = 46)	CKD4–5 (*n* = 23)	CKD5D (*n* = 23)
Phosphate binder	37 (80%)	21	16
Ca-based binders alone	35 (76%)	20	15
Calcium carbonate/calcium acetate	16/19	12/7	4/12
Ca-based binder and sevelamer	2 (4%)	1	1
Calcium supplements	7 (15%)	2	5
Calcium carbonate	5	0	5
Calcium glubionate and calcium lactobionate	2	2	0
Ca-based phosphate binder and Ca supplement	4 (9%)	2	2
Phosphate supplement	5 (11%)	0	5

**Fig. 4:**
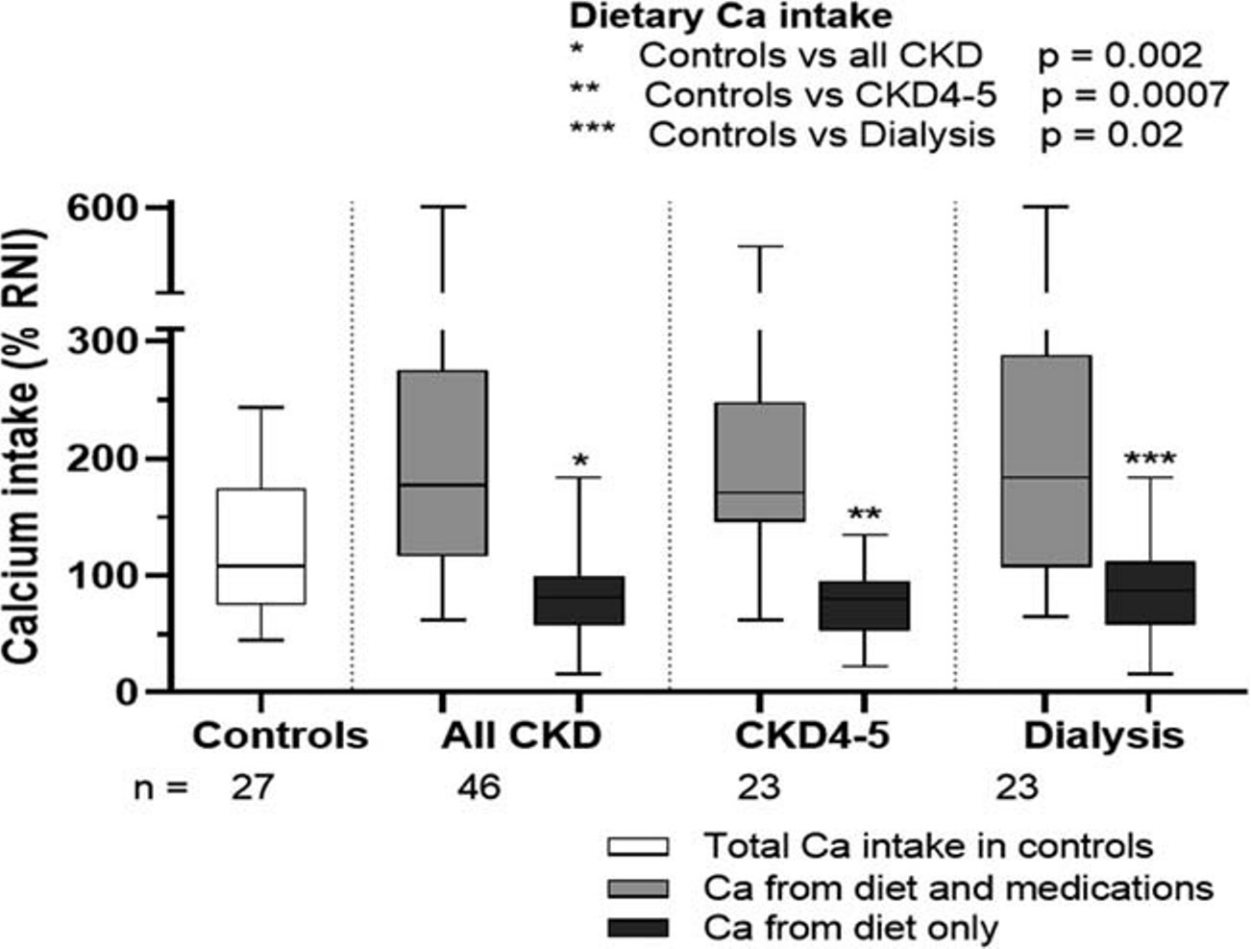
Ca intake from diet and medications expressed as a percentage of reference nutrient intake (% RNI) for age

The Ca intake from diet and all medications as % RNI is shown in Fig. 4. Including the Ca from medications increased the median total Ca intake to 177 (IQR 119–270) % RNI, medications contributing 59.5 (IQR 29.5–74.0) % of total daily Ca intake. For children with CKD4–5, the relative contribution from medications to total calcium intake was highest in those with the lowest eGFR (*p* = 0.01). In CKD4–5D patients, the total Ca intake was < 100% RNI in 15%, 100–200% RNI in 44% and > 2 × RNI in 41%. Three (6%) of the CKD4–5D patients exceeded the KDOQI recommended upper limit of 2500 mg Ca/day.

#### Ca intake and serum biochemistry

There were no significant differences in serum calcium between those exclusively FF and those not on FF. Serum Ca, ionized Ca, PTH, or alkaline phosphatase did not reflect dietary or total Ca intake. None of the 7 patients with a total Ca intake below RNI was hypocalcemic (albumin-corrected serum Ca < 2.0 mmol/L), and only 1 of the 19 with a total Ca intake > 2 × RNI was hypercalcemic (albumin-corrected serum Ca > 2.6 mmol/L). There was no relationship between vitamin D status and other measures of Ca status or bone health (serum Ca, ionized calcium, PTH, or alkaline phosphatase).

## Discussion

In this study, we have shown that Ca intake from diet alone was inadequate in 76% of children with CKD4–5D, median dietary Ca intake being 81% RNI for age, and significantly lower than that for age-matched controls. Phases of rapid growth in childhood and adolescence are critical periods for bone mass accrual [3], giving rise to a greatly increased Ca requirement [9]*.* Studies in healthy pre-pubertal children receiving calcium supplementation showed increase in bone mass and augmented bone response to physical activity compared with non-supplemented controls [21, 22]. High calcium intakes seem to be protective against fractures in healthy adolescents [23]. Our data support the KDIGO [10] and NICE [11] recommendations for the use of Ca-based P-binders as first-line treatment for hyperphosphatemia in children with CKD and on dialysis.

These findings are in agreement with the Growth Failure in Children with Renal Disease (GFRD) study [24], where children with CKD achieved a Ca intake of 80% of normal requirements for age. Other studies have described similar or even lower Ca intakes [25–27], even from CKD stage 3 [28]. It is likely that a poor appetite also contributes to the reduced Ca intake in those with CKD.

The relative contribution of different food groups to total dietary Ca intake in pediatric CKD has not been previously reported. The lower proportion of dietary Ca in CKD4–5D from dairy foods (40% vs 56% in controls), and the lower P intake assessed from the diet diaries, suggests that the reduction in dietary Ca intake may be an inadvertent consequence of advice to reduce P intake. Limiting dairy foods (along with avoidance of P food additives) often constitutes the first-line intervention for serum P control. In this cohort, all the children with CKD had been advised on dietary phosphate restriction. Excluding those exclusively on FF, cereals contributed 33% and 27% of dietary Ca intake in CKD and controls, respectively. Of note, mandatory fortification of most flour in the UK [29] (and the voluntarily fortification of foods such as breakfast cereals) increases the contribution of cereals to daily Ca intake in our patients. However, in countries where fortification is not common practice, the Ca intake in this population may be even lower. The contribution of FF to dietary Ca intake has not been previously studied. Children with CKD who were exclusively fed FF achieved a higher dietary Ca intake which was comparable with that of controls (*p* = 0.08).

A previous study reported a reduction in dietary Ca intake as renal function declines through all CKD stages [25]; we found this in those with CKD4–5, but not in those on dialysis, possibly due to the higher use of FF in the latter group. This finding emphasizes the importance of offering enteral tube feeding to improve overall nutrition (including Ca intake) in those struggling with a poor oral intake, as recommended by the Pediatric Renal Nutrition Taskforce (PRNT) [30]. Our results suggest a greater reliance on Ca-based medications with advancing CKD, perhaps as dietary P restrictions increase or are more strictly enforced, inadvertently reducing dietary Ca intake.

No previous dietary studies in children with CKD have quantified the contribution from Ca-containing medications. We have shown 76% of CKD4–5D patients received insufficient dietary Ca, and that Ca-based P-binders and Ca supplements improved Ca intake to a median of 177% RNI. While both KDIGO and NICE recommend Ca-based P-binders should be the first-line treatment for hyperphosphatemia in children with CKD [10, 11], the absorption of Ca from these binders is uncertain (reported to be between 20 and 30%). Absorption is generally lower than that from food, varies with the timing of administration in relation to food, and differs between Ca-carbonate and Ca-acetate [7, 31]. In addition, Ca absorption varies between 15 and 40% depending on the individual’s vitamin D status [32]. This variable absorption (which also applies to food-derived Ca) was not adjusted for in our analysis and has been identified as a future research need by the PRNT in their clinical practice recommendations for Ca and P for CKD2–5D [12].

Taking into account body size, dietary Ca intake, expressed as mg per kg body weight, decreased with increasing age in controls and children with CKD4–5D (as shown in Supplementary Fig.1). It is difficult to attribute any importance to this as Ca requirements are influenced by a number of factors that are dependent on age rather than weight. The reported difference in dietary Ca intake (as a % RNI) between controls and those with CKD4–5D was still significant when adjusted for height age.

RNI for Ca was not met by 44% of controls, suggesting that a low Ca intake in the general pediatric population may be a concern, particularly in those over 10 years of age. This finding was also reflected in the latest report from the NDNS, where an increasing proportion of UK children in the age group 11 to 18 years had an insufficient dietary Ca intake [33]. The wide variation in published international requirements for Ca [12] and the increased availability of data on Ca balance in children may suggest that the UK RNI figures which were published in 1991 are no longer an appropriate reference against which to benchmark Ca intake. This has been addressed by PRNT [12] who propose a new term, Suggested Dietary Intake (SDI), which reflects the range of recently published international requirements derived using the most reliable methods for assessing mineral status (and does not include the UK RNI). Only 11% of our CKD cohort had a dietary Ca intake within the SDI, compared with 24% achieving UK RNI as the latter is set lower for most age groups. Although international guidelines [10–12] recommend that clinical decisions are based on serum Ca (or ionized Ca) levels, none of the 7 children with a total Ca intake below RNI was hypocalcemic, and only one patient having > 2 × RNI was hypercalcemic, suggesting that serum Ca may not be a useful guide to the adequacy of Ca intake. The true Ca requirements for any individual depend on their age, growth, and rate of bone turnover [12]. To fully interpret our results, and understand the implications of high and low Ca intakes in children with CKD, careful Ca balance studies are needed to determine Ca absorption, state of bone mineralization, and fate of the ingested Ca. Meanwhile, as recommended by KDIGO, trends in Ca, P, PTH, and ALP, taken together, are the best available measure for the clinical management of MBD [10]. Indeed, as shown in a recent study in children and young adults with CKD 4–5D [34], tibial cortical bone mineral density (BMD) was negatively associated with parathyroid hormone (PTH; *r* = − 0.44, *p* < 0.001) and alkaline phosphatase (ALP; *r* = − 0.22, *p* = 0.03) and positively with serum calcium (*r* = 0.33, *p* = 0.001). On multivariable linear regression PTH, ALP and calcium together predicted 57% of variability in tibial cortical BMD measured by peripheral quantitative CT scan, whereas dual energy X-ray absorptiometry did not correlate with biochemical measures.

Forty-one percent of our CKD4–5D cohort received > 200% RNI for Ca as a result of the contribution from Ca-based P-binders. However, in this study, we did not record adherence to medications and this may have resulted in an overestimation of total Ca intake, as adherence to P-binder medication is notoriously poor [35, 36]. It is also not certain how much calcium is absorbed from these binders [7]. In clinical practice, in the presence of high serum calcium, we would carefully check the patient’s dosage and timing of administration of Ca-based P-binders in relation to their dietary phosphate intake. In our experience, patient education often results in a lower overall dosage, when binders are being used unnecessarily with low phosphate meals. In addition, patients would be instructed not to give calcium carbonate at the same time as ranitidine or other H2 blocker as gastric acidity is required for the dissociation of calcium carbonate and its phosphate-binding effect [7].

Limitations of our study include the incompleteness of the diet diaries (3 CKD4–5D and 3 controls completed only 2 rather than 3 days). The use of a semi-quantitative prospective 3-day diet diary is considered to be an appropriate dietary assessment tool for Ca or P intake [12, 37], but imposes a high participant burden with possibly incomplete or selective recording [37, 38]. In an attempt to limit potential errors due to coding and interpretation of the diaries, a single dietitian performed all the analyses. Nutrient databases and dietary assessment software packages to calculate Ca intake are considered reasonably accurate when compared with direct chemical analyses [38], but a food database does not fully account for Ca intake from food additives, food fortification, or from drinking water. Ca intake from dialysate was also not included in the Ca analysis.

In conclusion, 76% of the CK4–5D study population from a large pediatric tertiary care hospital had a dietary Ca intake below 100% RNI for age. A reduction in Ca intake from dairy foods is likely to account for this. In our cohort, 80% of patients had Ca-based P-binders and 9% also had Ca supplements, increasing Ca intake to 177% RNI and thus meeting the KDOQI guidelines for CKD of 100–200% recommended intakes for the normal population. Just as with healthy children, an adequate Ca intake is required to meet the needs of the growing skeleton in children with CKD.

## Electronic supplementary material

ESM 1 (DOCX 24 kb)

ESM 2 (PPTX 93 kb)

Supplementary Fig. 1 Dietary Ca intake expressed as mg/kg body weight for controls and CKD4–5D. The linear regression with 95% confidence intervals is shown (PNG 565 kb)

High resolution image (TIF 105 kb)
