# Supplementary material for: Dietary calcium intake does not meet the nutritional requirements of children with chronic kidney disease and on dialysis
Source: Pediatr Nephrol. 2020 May 8;35(10):1915–23. doi: 10.1007/s00467-020-04571-x (PMC7501104; doi:10.1007/s00467-020-04571-x)
Supplement: Supplementary file 1 — (DOCX 24 kb) [file 467_2020_4571_MOESM1_ESM.docx]

# Dietary calcium intake does not meet the nutritional requirements of children with chronic kidney disease and on dialysis

# – Supplementary material

**Results:**

**P intake in children with CKD4-5D and controls**

The median P intake of children with CKD4-5D was 610mg/day, 125% RNI, and significantly lower than controls who received 982mg (172% RNI; p=0.00008; Supplementary Table 1). Of note, this assessment likely underestimates total P intake in both populations, as the contribution from P additives is unknown. Five of the 23 children on dialysis (22%) were receiving P supplements providing between 47 and 62mg additional P per day, four being on in-center hemodialysis and one home hemodialysis. None of those with CKD4-5 had P supplements.

In controls, the distribution of sources of P was similar to NDNS average data 4-18 years (14,15), with over one third (36%) coming from dairy foods and egg, 28% from cereals and cereal products and about 20% from meat and meat products (Supplementary Table 2). For all groups (controls and CKD) dairy, cereals and meat provided more than half of their P intake. The significant contribution of dairy and cereals to both Ca and P intakes is reflected by the close correlation between Ca and P intake (as % RNI) in the children with CKD4-5D (p < 0.0001, R^2^ = 0.51; Figure 3).

**Supplementary Table 1** Phosphate intake (% RNI; median [IQR])

#####

| **GROUP** | **Dietary P (% RNI)** |
| --- | --- |
| **All CKD (CKD4-5D)** | 125 (100-159) |
| **CKD4-5** | 132 (108-157) |
| **CKD5D** | 110 (96-158) |
| **CKD (exclusively formula fed)** | 99 (64-124) |
| **Controls** | 172 (129-238) |

**Supplementary Table 2** Percentage contribution of food groups to dietary P intake

| **FOOD GROUP** | **UK**  **reference values** | **Controls**  (n=27) | **CKD4-5D**  Diet and formula feeds  (n=46) | **CKD4-5D**  Diet only  (n=34) |
| --- | --- | --- | --- | --- |
| **Formula feeds** | - | - | 30 | - |
| **Dairy and egg** | **25-36** | 36 | 16 | 20 |
| **Cereals and cereal products** | **24-27** | 28 | 20 | 28 |
| **Meat and meat products** | **15-20** | 20 | 17 | 27 |
| **Other** | **19-21** | 16 | 17 | 25 |

***UK reference values** [14, 15]
