# Supplementary material for: Dietary calcium intake does not meet the nutritional requirements of children with chronic kidney disease and on dialysis
Source: Pediatr Nephrol. 2020 May 8;35(10):1915–23. doi: 10.1007/s00467-020-04571-x (PMC7501104; doi:10.1007/s00467-020-04571-x)
Supplement: Supplementary file 2 — (PPTX 93 kb) [file 467_2020_4571_MOESM2_ESM.pptx]

## Slide 1
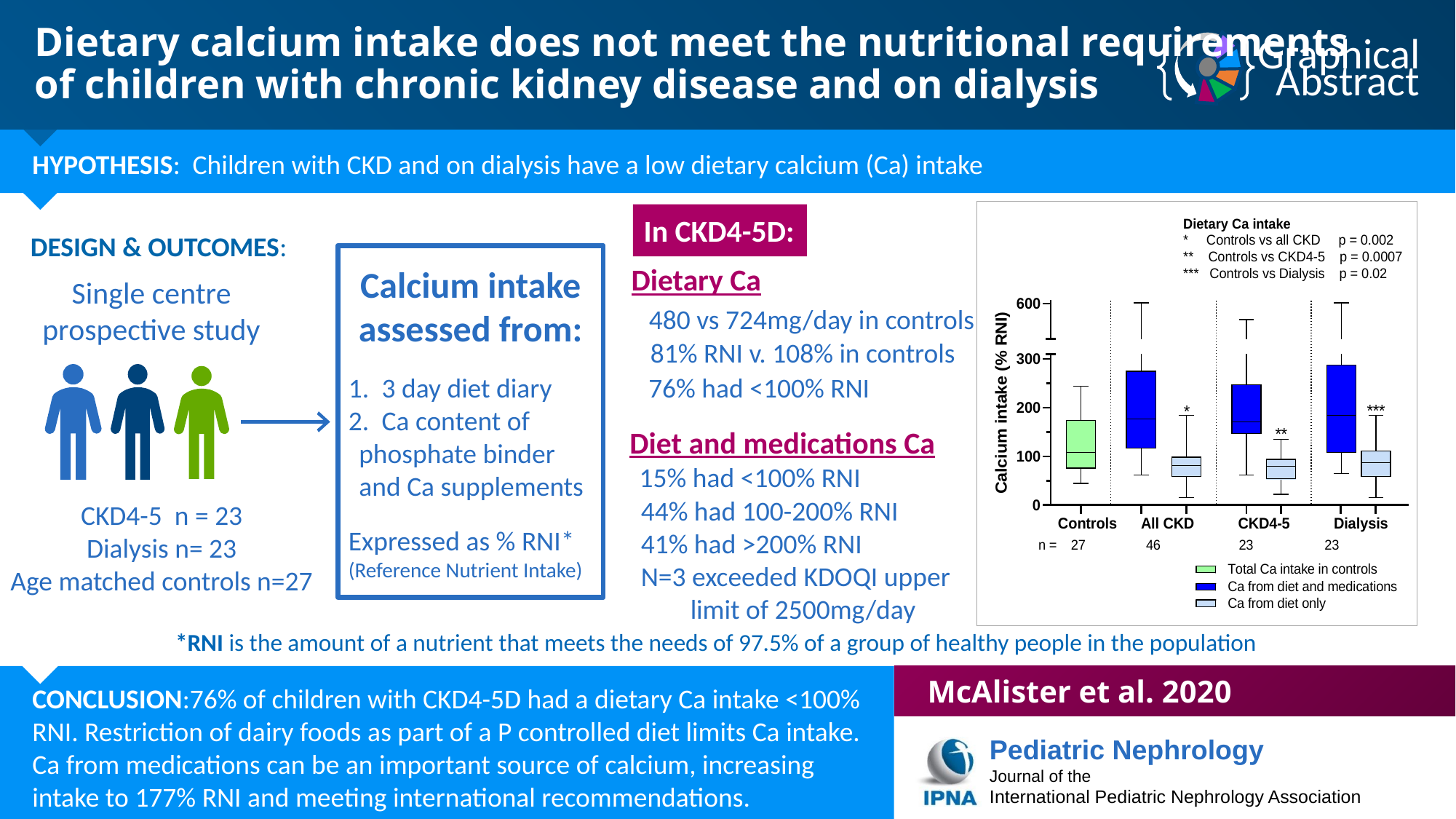

Dietary calcium intake does not meet the nutritional requirements
of children with chronic kidney disease and on dialysis
HYPOTHESIS: Children with CKD and on dialysis have a low dietary calcium (Ca) intake
In CKD4-5D:
DESIGN & OUTCOMES:
Calcium intake assessed from:
 3 day diet diary
 Ca content of phosphate binder and Ca supplements
Expressed as % RNI* (Reference Nutrient Intake)
Dietary Ca
Single centre prospective study
480 vs 724mg/day in controls
81% RNI v. 108% in controls
76% had <100% RNI
Diet and medications Ca
15% had <100% RNI
44% had 100-200% RNI
41% had >200% RNI
N=3 exceeded KDOQI upper
 limit of 2500mg/day
CKD4-5 n = 23
Dialysis n= 23
Age matched controls n=27
*RNI is the amount of a nutrient that meets the needs of 97.5% of a group of healthy people in the population
McAlister et al. 2020
CONCLUSION:76% of children with CKD4-5D had a dietary Ca intake <100% RNI. Restriction of dairy foods as part of a P controlled diet limits Ca intake. Ca from medications can be an important source of calcium, increasing intake to 177% RNI and meeting international recommendations.
