# Supplementary figures and images for: Dietary calcium intake does not meet the nutritional requirements of children with chronic kidney disease and on dialysis
Source: Pediatr Nephrol. 2020 May 8;35(10):1915–23. doi: 10.1007/s00467-020-04571-x (PMC7501104; doi:10.1007/s00467-020-04571-x)

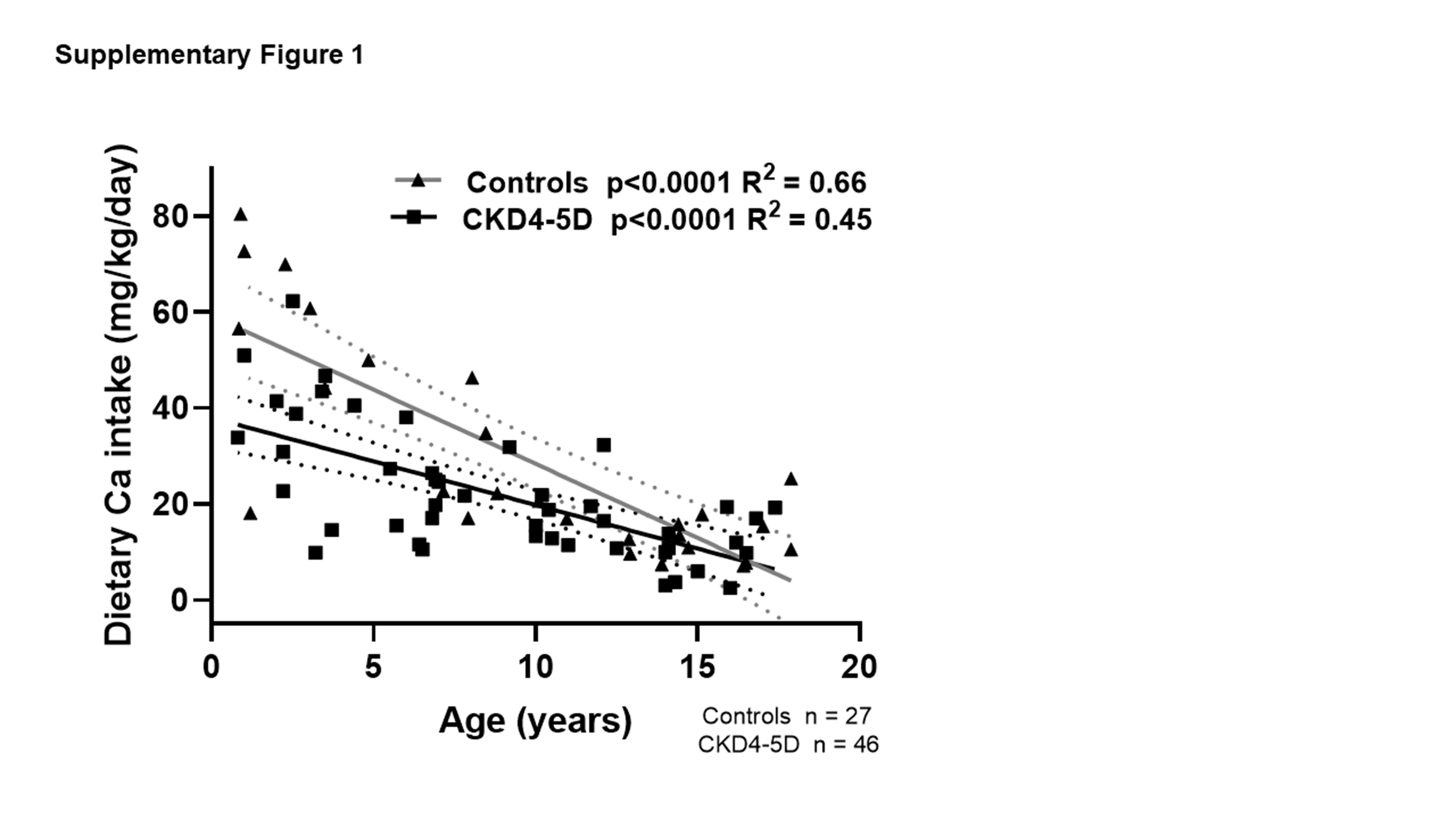

Supplement: Supplementary file 3 — Dietary Ca intake expressed as mg/kg body weight for controls and CKD4–5D. The linear regression with 95% confidence intervals is shown (PNG 565 kb) [file 467_2020_4571_Fig5_ESM.png]

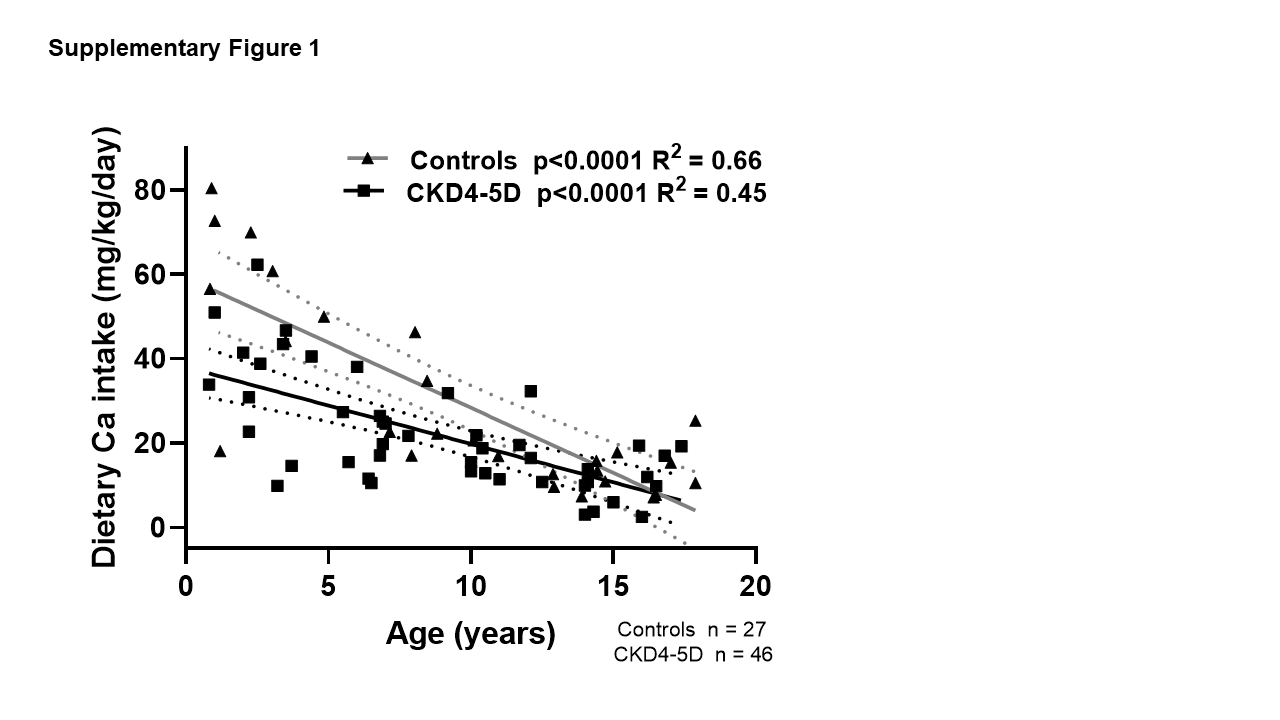

Supplement: Supplementary file 4 — High resolution image (TIF 105 kb) [file 467_2020_4571_MOESM3_ESM.tif]
